# Development and characterization of novelly grown fire-resistant fungal fibers

**DOI:** 10.1038/s41598-022-14806-6

**Published:** 2022-06-27

**Authors:** Xijin Zhang, Yanjun Li, Xudong Fan, Gary Wnek, Ya-Ting T. Liao, Xiong Yu

**Affiliations:** 1 Department of Civil and Environmental Engineering, Case Western Reserve University, Cleveland, OH 44106 USA; 2 Department of Mechanical and Aerospace Engineering, Case Western Reserve University, Cleveland, OH 44106 USA; 3 Department of Macromolecular Science and Engineering, Case Western Reserve University, Cleveland, OH 44106 USA

**Keywords:** Microbiology, Engineering, Materials science

## Abstract

This study conducted a comprehensive characterization and analyses on the fire-resistant behaviors of novel fungal fibers grown with substrate containing Silica (Si) source at multiple scales. At micro-scale, the results of SEM showed that silica affected the physiological activities of fungi, with the extent of effects depending upon its concentration. Fourier-transform infrared (FTIR) spectra displayed the existence of Si–O–C chemical bonds in fungal fibers grown with Si source, indicating that Si source becomes a part of the structure of fungal fibers. Thermogravimetric analysis (TGA) and Microscale combustion calorimetry (MCC) of fungal fibers exhibit an early thermal decomposition of non-combustible components, which will potentially help release the thermal stress and mitigation of spalling when used in concrete. Compared with polypropylene (PP) fibers, fungal fibers have a lower thermal degradation rate, a higher residual weight, a lower heat release peak temperature, and less total heat of combustion; all of these indicate improved thermal stability and fire resistance, and a lower rate of function loss in case of a fire. Additionally, the thermal stability and fire resistance of fungal fibers were improved with the increase of Si source concentration in the nutrition medium. For example, addition of 2% Si source in the feeding substrate leads to a 23.21% increase in residual weight in TGA, and a 23.66 W/g decrease in peak heat release rate as well as a 2.44 kJ/g reduction in total heat of combustion in MCC. At laboratory scale, compared with PP fibers, fungal fibers grown with 2% Si source have a higher residual weight of 40.40%, a higher ignition temperature of 200.50 °C, and a declined flame height of 11.64 mm in real fire scenarios. Furthermore, only in the fungal fibers grown with Si source, partial burning occurred. In post-fire conditions, the microstructure of residual char from fungal fibers grown with higher content of Si source became denser, which would lead to a reduction of the fuel vapor release and heat transfer. FTIR spectra of residual char demonstrated that fungal fibers grown with Si source formed more stable chemical bonds with higher heat of chemical bond formation, contributing to improved thermal stability and fire resistance. Therefore, compared with traditional fibers used for fiber reinforced concrete, incorporating the new natural grown fibers will potentially further improve the fire resistance of concrete and mitigate the concrete spalling.

## Introduction

With the increasing threat of fire on residential buildings, fire safety becomes a key consideration in building design. Using fire-resistant materials and products in buildings can help prevent and control potential fire accidents, decreasing the property damage and reducing civilian deaths and injuries^1,2^. Concrete structures have the risk of spalling when subjected to fire, which results from the accumulation of vapor pressure^3,4^. The spalling of concrete exposes deeper layers of concrete to fire and heat propagation, potentially reaching the embedded reinforcement bars. Exposure to fire also compromises the mechanical strength of concrete^5^.

Commercial fibers have been commonly utilized to improve the tensile strength of concrete and to attenuate the spalling of concrete^6,7^. High strength concrete is more susceptible to the accumulation of vapor pressure due to its dense structure and low permeability^8^. The melting of fibers starts at low temperature, which serves as passage for water vapor to escape and therefore mitigated the spalling damage when subjected to heating^6,7^. However, thermal decomposition of fibers inevitably leads to the failure of their bridging ability, which eventually causes the loss of mechanical strength in the reinforced concrete. The melting and degradation of fibers were found to be contributors to the significant reduction in residual strength of Polypropylene (PP) fiber reinforced concrete^5,9^. Previous studies have indicated that the faster degradation of fiber reinforced concrete as temperature rises was responsible for its lower fire resistance as compared with normal concrete structure^10^. Çavdar found that the use of noncombustible carbon fibers retained the highest compressive strength compared with Polyvinyl alcohol (PVA) and Polypropylene (PP) fibers^8^. Additionally, epoxy resin has been usually used to improve the bonding strength in the conventional fiber reinforced concrete. However, epoxy resin had poor fire resistance and it degraded at 70 °C. It has been proven that the fire accidents especially reduced the bearing capacity of fiber reinforced concrete with use of epoxy resin significantly^6^. In general, the early melting of fibers mitigates spalling of concrete, however, quick degradation of fibers leads to a high rate of function loss in concrete; fibers eventually lose the majority of their weight, weakening the concrete. Moreover, using synthetic fibers and epoxy resin has several drawbacks, including high cost, poor recycling, and unsustainable properties^11^.

With the increasing threat to buildings posed by fire incidents and the mounting pressure to reduce pollution in the manufacturing processes, the pursuit of alternative natural fibers that are more fire resistant and less polluting has become a significant objective of recent studies. Naturally grown fungal fibers have already been proven alternatives to synthetic fibers in construction and packaging industries due to their low density, satisfactory mechanical strength, and low-cost property. The dry fungal fiber-based material showed a lighter weight than water and most other materials used in the field of Civil Engineering^12^. Moreover, mycelium grown by *Ganoderma lucidum* showed ductile property with an elongation of 33% at break, which was less brittle compared with two other biopolymers, i.e., bacterial cellulose and polyhydroxyalkanoates^13^. The fungi-based composites exhibited a rupture modulus of 4.6 MPa, an elasticity modulus of 680 MPa, and an internal bonding strength of 0.18 MPa after heat pressing at 200 °C^14,15^. Additionally, while fungi mycelium-based composites share similar mechanical properties with commercial Styrofoams (i.e., expanded polystyrene, polyethylene, polyurethane and wool), the fungi have a much lower cost^16,17^. Therefore, fungi mycelium-based composites have been utilized building construction^18^. Recent studies demonstrate that fungal fibers also exhibited exclusive thermal stability and fire resistance. Fungal fibers from *Pleurotus ostreatus* and *Fusarium oxysporum* retained an increasing residual weight of 2122% and 2223% at 800 °C respectively when compared with PVA fibers^19^. Additionally, Si-rich nutrition was absorbed by fungi and was incorporated into their fibers’ structure, improving thermal properties. The post-fire residual weight of *F. oxysporum* grown with nutrition containing 3% (volume fraction) Si source was improved by 93.81% at 800 °C in the thermogravimetric analysis (TGA)^19^.

While previous studies investigated the potential of cultivating fungi with Si source to improve thermal resistance, there is a lack of data on the comprehensive fire-resistant properties of fungal fibers in real fire scenarios, especially the characteristics during fire and under post-fire conditions. Such data is essential for evaluating fire performance before applying them to the concrete structural member, which is a basic design requirement in building. Aligned with this motivation, a series of experiments at micro-scale and laboratory-scale, i.e., the thermogravimetric analysis (TGA), the microscale combustion calorimetry (MCC), the upward flame spread test, and the heating ignition test, were carried out to characterize the fire-resistant behaviors of fungal fibers grown with Si source. The morphology and chemical compositions of post-fire residuals were also investigated. The fire resistance behaviors of fungal fibers grown with Si source was compared with that of polypropylene (PP) fibers that are commonly used in concrete. The results indicate that fungal fibers grown with Si source show superior fire-resistant behaviors.

## Materials and methods

### Fungal strain

The filamentous fungus strain, *F. oxysporum* (ATCC MYA-1198), with a biosafety level of 1, was chosen for this study. This fungus is not harmful to humans and features a high growth rate.

### Substrate preparation

The Potato Dextrose Agar (PDA) powder was provided by Fisher Scientific (DF0013-17-6). The Si source, silica sol–gel, was obtained from the Sigma-Aldrich Company (338,443-1L). The culture medium was prepared by dissolving PDA into deionized water with a concentration of 39 g/L. The Si source was added into the culture medium at different volume fractions after being autoclaved. The PP fabric was purchased from Berkshire (Item # PW750.1212.20).

### Contrast groups of experimental samples

Three contrast groups of experimental samples were included in the study, i.e., fungi grown with a substrate without PP fabrics, fungi grown with a substrate with PP fabrics, and PP fabrics submerged in a substrate without the inculcation of fungi. For the substrate used for each group of samples, different concentrations of Si source, 0%, 1%, 2%, 3%, and 5%, were added to the PDA culture media. The summary of the experiment components is shown in Table 1.

**Table 1:** Contrast groups of experimental samples.

Sample Name*	Substrate constitutions			Inoculum
	PDA	Si contents	PP Fabric	Fungi
FSi0	√	0%	×	√
FSi1	√	1%	×	√
FSi2	√	2%	×	√
FSi3	√	3%	×	√
FSi5	√	5%	×	√
FSi0-P	√	0%	√	√
FSi1-P	√	1%	√	√
FSi2-P	√	2%	√	√
Si0-P	√	0%	√	×
Si1-P	√	1%	√	×
Si2-P	√	2%	√	×

Annotation: × : without; √: with.

*Name convention: F represents *F. oxysporum*, Si represents the Si source, the number represents the volume fraction percentage of Si source, P represents PP fabrics.

All the materials were sterilized at 120 °C for 15 min prior to use. PP fabrics were cut and placed at the bottom of Petri dishes with an inner diameter of 86 mm. 15 mL of the substrate with different concentrations of Si source were placed in Petri dishes. *Fusarium oxysporum* with a diameter of 5.75 mm was obtained from the leading edge of the original colony by using a sterile cork borer. Fungal inoculums were inoculated at the center of the substrate in the Petri dishes. The Petri dishes were covered with oversized lids (diameter offset: 2.5 mm) during the incubation period. This introduces oxygen, enabling fungal growth and preventing contamination. All the samples were cultivated at controlled temperature (20 °C ± 2 °C) and humidity (35% ± 3%).

### Experimental procedure

This paper aims to characterize the fire resistance of fungal fibers grown with Si-rich nutrition at both micro-scale and laboratory-scale and compare their fire-resistant performance with that of PP fibers. Comparison the flammability of different materials is difficult. Therefore, the fungi were inoculated on the surface of PP fabrics to form an interaction of PP fibers and fungal fibers (fungi-PP fabric). The speed at which the flame spread on the fungi-PP fabric was compared with untreated PP fabric to identify whether the existence of fungal fibers accelerated or inhibited the spread of the flame on PP fabrics.

**Figure 1:**
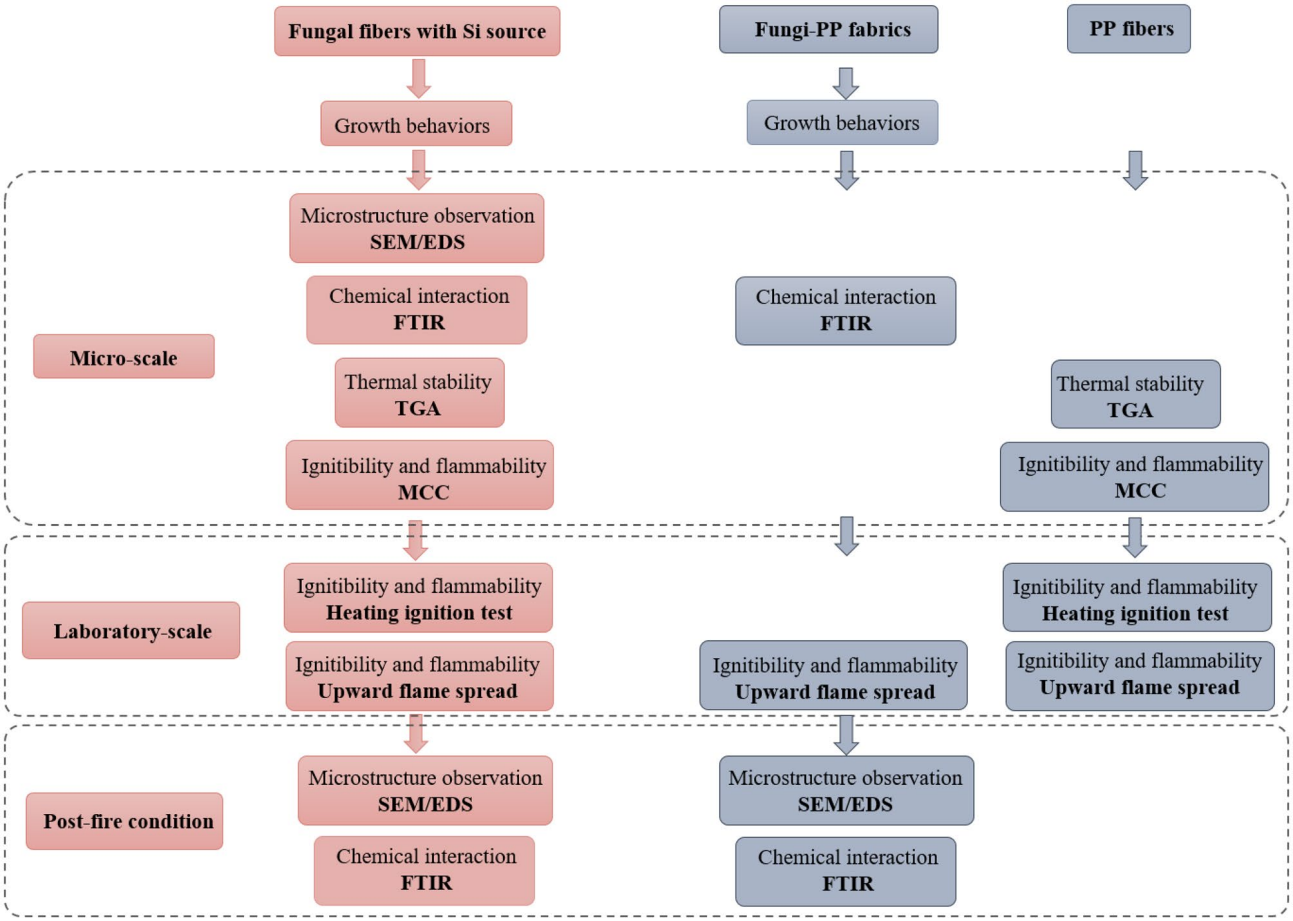
Scheme of experimental design analyzing the multi-scaled behaviors of contrast samples.

The first stage of this study focused on the growth behaviors of fungal mycelium grown with different substrates. Then, the influence of Si source and PP fabric on the development of the mycelium were investigated. In the second stage of the study, the microstructure, chemical interaction, and fireproofing properties of the fungal fibers were evaluated at the micro-scale. At the laboratory-scale, the third stage of the study, a heating ignition test and upward flame spread test were utilized to explore the ignition temperature and flame spread speed of fungal fibers. In the fourth and final stage of the study, the microstructures and chemical composition of the samples in post-fire conditions were also analyzed. The flow chart of experimental procedures in this study is summarized in Fig. 1.

#### SEM and EDS analysis

An elemental microanalysis technique, scanning electron microscopy with energy dispersive X-ray spectrometry (SEM/EDS) was applied to observe the microstructure and element distribution of each sample. SEM characterization was performed by a Helios NanoLab 650 with a voltage of 5 kV and EDS analysis was conducted with a high voltage of 20 kV and a current of 1.6 nÅ.

#### Fourier-transform infrared

Fourier-transform infrared (FTIR) spectra were measured using an Agilent Technologies Cary 630 FTIR spectrometer equipped with a diamond crystal detector using transmission geometry. All of the FTIR spectra were recorded by integrating 900 scans with a range from 400 to 4000 cm^-1^.

#### Thermogravimetric analysis

Thermogravimetric analysis (TGA) was conducted by TA Instruments (TGA Q500) in an airflow. Samples were dried in an oven at 50 °C for 24 h prior to the test. Fungal fibers grown with substrates containing various concentrations of Si source with 5–10 mg were placed in a platinum pan without a lid. The samples are heated at a rate of 10 °C/min. Their mass changes were recorded as they were heated in temperatures ranging from 100 to 800 °C.

#### Microscale combustion calorimetry

Microscale combustion calorimetry (MCC) was developed by the Federal Aviation Administration to evaluate the ignitability and flammability of materials. MCC tests consist of the pyrolysis, combustion gas, and flow calorimetry, which is performed by pyrolysis-combustion flow calorimetry (PCFC). A sample of 3.5–5 mg of PP or fungal fibers was placed in the nitrogen flow at 80 ml/min in a pyrolyzer that was heated up to 800 °C with a heating rate of 10 °C/min. The gaseous products of the pyrolysis were carried by the nitrogen flow into the combustor that was filled with oxygen flow at 20 ml/min. The combustion heat release rate of the pyrolysates was analyzed based on the recorded oxygen concentration consumption rate.

#### Heating ignition test

**Figure 2:**
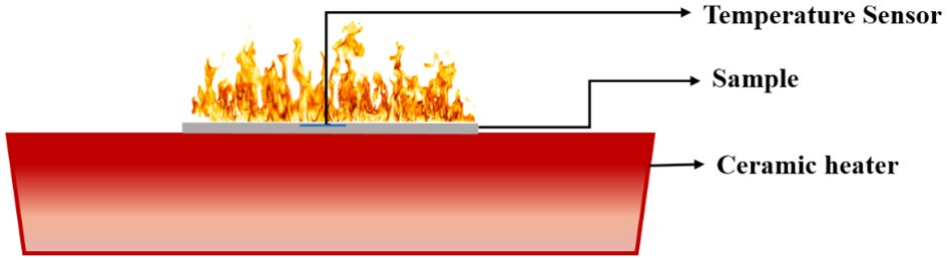
The schematic diagram of ignition temperature test.

Heating ignition test was carried out by applying a constant heat flow on the samples. Schematic of the test setup is shown in Fig. 2. In this process, thin films generated by the growing of fungal fibers and thin PP fabrics were placed on a radiant ceramic heater. The heater was heated up and reached a steady heat flow prior to the test. A metallic grid (5 mm) was positioned above the heater. The heat flow at the center of the metallic grid was measured using the PHFS-005 heat flux sensor (Flux Teq Inc.). Once the sensor approached the measurement position, the heat flow quickly increased and reached a constant value. After nine scans, the ignition heat flux was measured as 17.1 ± 0.49 kW/m^2^. During the tests, the sample was quickly placed onto the metallic grid and was heated by the steady heat flow. A thermocouple with a diameter of 0.127 mm (Omega Engineering Inc.) was sewed onto the surface of the sample to measure the ignition temperature. The initial and final weight were also measured, and the maximum flame height was determined using image analysis. Each test was repeated nine times to mitigate variations in the samples.

#### Upward flame spread test

An upward flame spread test modified from UL-94 V^20^ was conducted. Samples were dried in the oven at 50 °C for 24 h prior to the test. Specimens were cut into pieces 65 mm (± 2 mm) long and 12.5 mm (± 0.5 mm) wide. The specimen was mounted vertically and clamped at the top. A Bunsen burner with a diameter of 11 mm was calibrated to generate a 20 ± 2 mm natural gas flame (around 2 W). The ignition of the specimens was achieved by placing the Bunsen burner 10 mm beneath the lower edge of the specimens. During the test, the specimen was exposed to the flame for 5 s. The ignition time was recorded, and the duration of the flame was recorded after the removal of the burner flame. The initial mass and residual mass of the specimens were also measured by a microbalance with a 0.10 mg scale. Each test was repeated nine times to account for differences in fungal fiber growth.

## Results and discussion

### The growth behavior of *Fusarium oxysporum*

**Figure 3:**
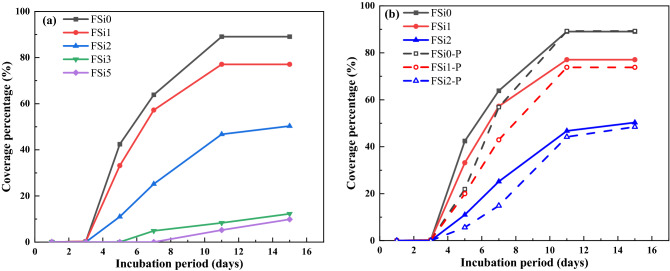
(**a**) Mycelium growth of *F. oxysporum* grown with different concentrations of Si source; (**b**) Comparison of mycelium growth between *F. oxysporum* grown with PP fabrics and without PP fabrics feeding on the same substrate.

The growth behaviors of *F. oxysporum* feeding on multiple substrates at different concentration levels of Si source were evaluated. Images were taken of each sample after different incubation periods to record the development of mycelium. The influence of adding both PP fabrics and Si source to the feeding substrate on fungal mycelium growth was also considered in the study. Photos of the growth patterns are summarized in Table 2 and the corresponding surface area coverage is plotted in Fig. 3. The surface area of fungal mycelium was analyzed via images by the open-source software Image J (https://imagej.nih.gov/ij/). The first section of the table includes all fungal mycelium grown with substrate containing different concentrations of Si source, i.e., FSi0, FSi1, FSi2, FSi3, and FSi5. Fungal inoculum germinated within the first 3 days in FSi0, FSi1, and FSi2. As the Si source was increased, it took longer for fungal inoculum to germinate, e.g., 4–5 days in FSi3, and 6–7 days in FSi5. Using the surface area of the Petri dish as a baseline, the percentage of surface area of the petri dish covered by fungal mycelium in each sample are 89.07%, 77.07%, 50.31%, 12.26%, and 9.85% after 15 days, respectively. This indicates that the absorption of Si source delayed the surface area development of fungal mycelium. The delay is a result of the addition of Si source that promotes the activities of lysosome, releasing more lytic enzymes. If the concentration of Si source is too high, the number of lytic enzymes that is produced will dissolve a large proportion of the fungal cell wall. In such a case, the normal activities of fungal cells, such as fungal growth, are inhibited^19^. The inhibiting effect was more obvious in the fungal fibers grown with higher concentration of Si source. The growth rates of fungal mycelium in FSi3 and FSi5 were so slow that they were not considered in the following laboratory-scale fire resistance tests.

**Table 2:**
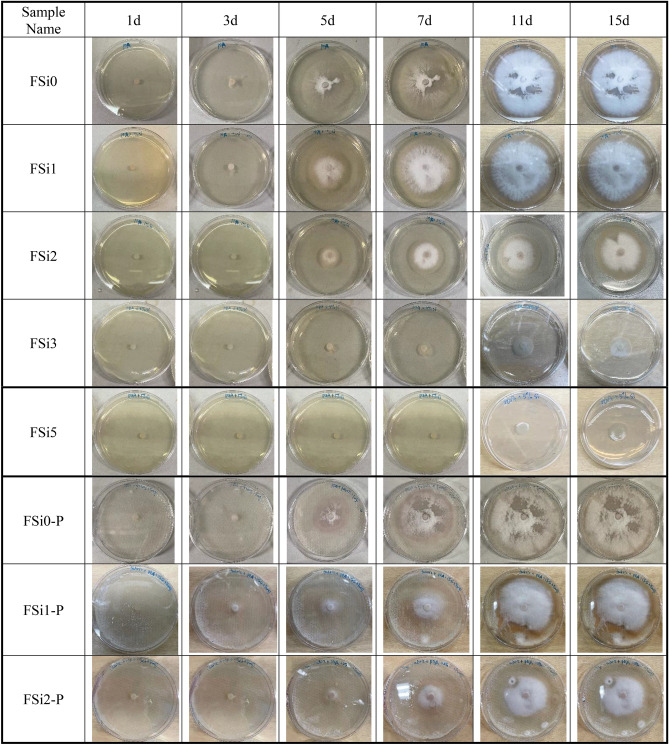
The growth behaviors of *F. oxysporum* grown with various substrates.

After 15 days, the surface area of *F. oxysporum* feeding on the Si-free substrate on PP fabrics (89.27% of Petri dish) was similar to that of *F. oxysporum* grown without PP fabrics. However, the surface area of fungi grown with 1% and 2% Si source on PP fabrics covered 73.83% and 48.46% of their respective Petri dishes, both of which are smaller than that of fungi grown without PP fabrics (covered 77.07% and 50.31% of Petri dishes, respectively). Therefore, it can be concluded that the addition of PP fabrics delayed the fungal mycelium development when feeding on substrate with 1% and 2% Si source; the influence of PP fabrics on fungal growth feeding on Si-free substrate is insignificant. PP fabrics was found to slightly delay the fungal growth, possibly because when the PP fabrics were placed in the feeding substrate, they physically may have influenced the distribution of the feeding substrate as well as the fungal absorption of nutrient.

### Micro-scale characterization

#### Microstructure of fungal fibers grown with Si source

**Figure 4:**
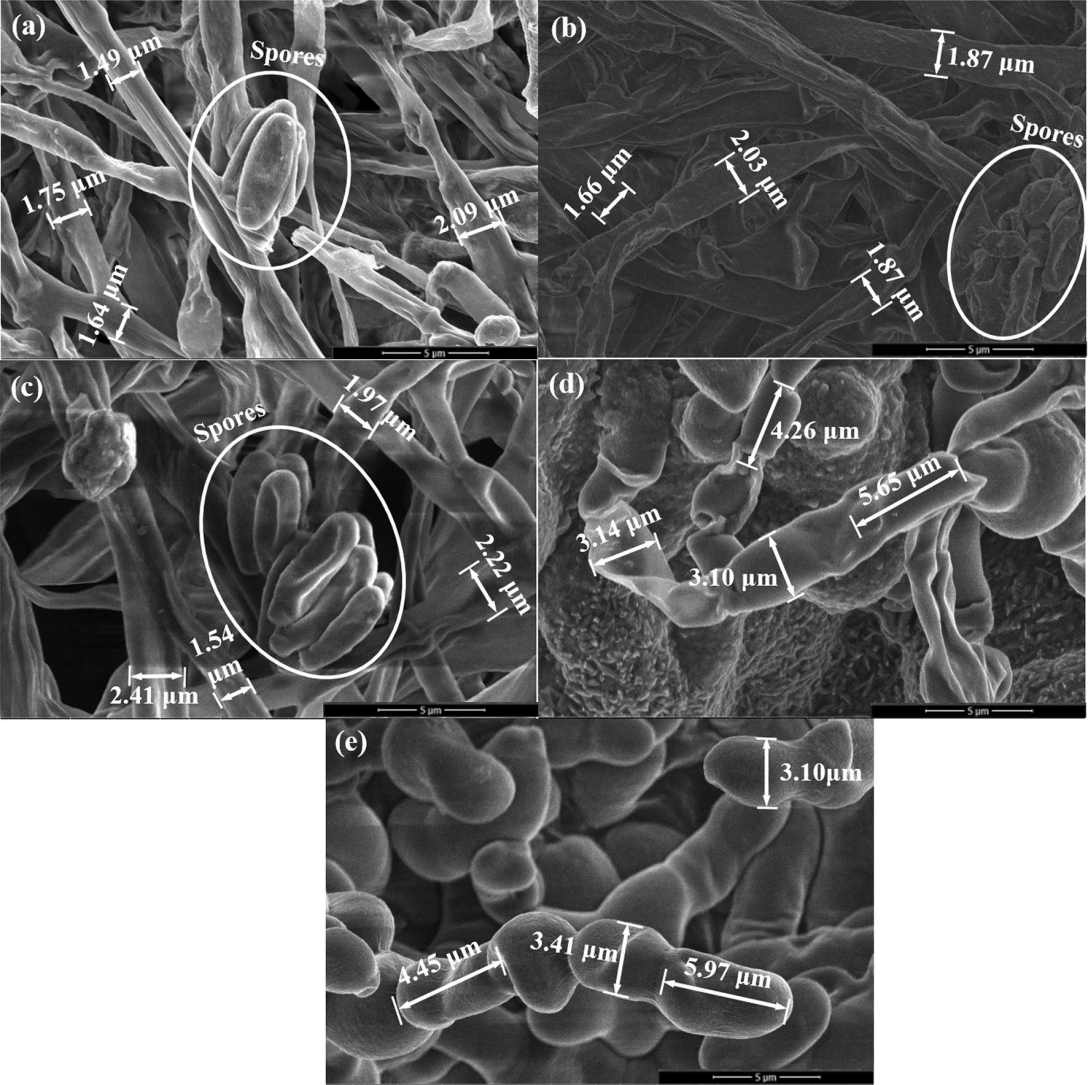
The microstructure of fungal fibers grown with Si source (**a**) Si-free substrate; (**b**) 1% Si source; (**c**) 2% Si source; (**d**) 3% Si source; (**e**) 5% Si source.

Scanning Electron Microscope (SEM) analysis was used for examining the morphological structures of fungal fibers grown with Si source. The addition of Si source generated a significant impact on the microstructures of the fungal fibers. The surfaces of fungal fibers grown with 0%, 1%, and 2% Si source (Fig. 4a–c) were straight and continuous. Several spores were found on the fungal fibers’ surface. The diameters of fungal fibers grown with 0%, 1%, and 2% Si source (Fig. 4a–c) were similar and ranged from 1.49 to 2.41 μm. However, fungal fibers grown with 3% and 5% Si source (Fig. 4d,e) were not straight and had a segmented structure. The lengths of distinct sections were around 4.26 μm to 5.97 μm. The diameters of fungal fibers grown with 3% and 5% Si source (Fig. 4d,e) were also much larger, ranging from 3.10 to 3.41 μm. The spores were not widely detected throughout the surface of the fungal fibers, which causes the lower growth rate in the sample with 3% and 5% Si source. These observations are directly associated with fungal metabolism. Once ingested into fungal cells, Si ions are essential for fungal metabolism^21^. Si source promotes the activity of lysosomes that discharge lytic enzymes^22^. Lytic enzymes make the cell wall plastic and easily elongated^22^. However, if Si source concentration levels are too high, fungal lysosome would release too many lytic enzymes, which dissolve a large proportion of the fungal cell wall. The plastic fungal cell walls might explain why the structure of fungal fibers become not straight and segmented when grown with 3% and 5% Si source. In addition, dissolving large part of fungal cell wall inhibits fungal growth, reducing visible spores in SEM images.

The EDS analysis was conducted on each sample spot indicated in Fig. 4. The weight ratios of corresponding main elements were summarized in Table 3. The fungal fibers grown with Si-free substrate (Sample a, FSi0) had a Si content of 0.12%. Slight increases of Si elements were found in fungal fibers grown with 1% and 2% Si source (i.e., with Si contents of 0.35% and 0.58% respectively). However, relatively higher Si contents (2.55% and 6.60%) were found in samples d and e (FSi3 and FSi5). Based on the mechanism of fungal growth with Si source, Si source would be absorbed in the fungal cell and incorporated as part of the fungal structures. Normal fungal activities and growth are inhibited due to the high concentration of Si source. This high concentration of Si source is the absorption threshold for fungi^19^. When fungi feeds on substrate containing Si source above the absorption threshold, the fungal cell wall dissolves and the structure of the fungal fibers becomes unsmooth^19^. Both SEM and EDS analyses indicate that 3% Si source is likely the absorption threshold of *F. oxysporum*. Under the threshold, most Si source were absorbed into fungal cells and only small amount of Si element (0.12 ~ 0.58%) were detected outside the fungi cells in FSi0, FSi1, and FSi2. Above the absorption threshold, higher concentration of Si source remained outside of the fungal cells, as seen in FSi3 and FSi5. High concentration of Si source leads to the changes in the fungal structure as well as to the reduction of fungal fiber production rates.

**Table 3:** The weight ratio of main elements in sample (a), (b), (c) and (d) in Fig. 3.

Sample ID	Si content	C	O	N	Na	Mg	Al	P	S	Cl	K	Ca	Si
a	0%	56.49	13.91	6.10	3.03	0.60	0.20	5.80	1.94	2.20	8.24	1.28	0.12
b	1%	65.37	23.51	–	2.38	0.14	–	1.98	0.48	0.94	4.86	–	0.35
c	2%	67.39	21.25	–	2.65	0.17	–	2.01	0.46	0.89	4.62	–	0.58
d	3%	62.17	27.87	–	5.99	–	0.09	0.63	0.18	0.21	0.32	–	2.55
e	5%	43.97	42.20	–	6.22	–	–	0.15	0.25	0.26	0.31	0.05	6.60

#### FTIR analysis

**Figure 5:**
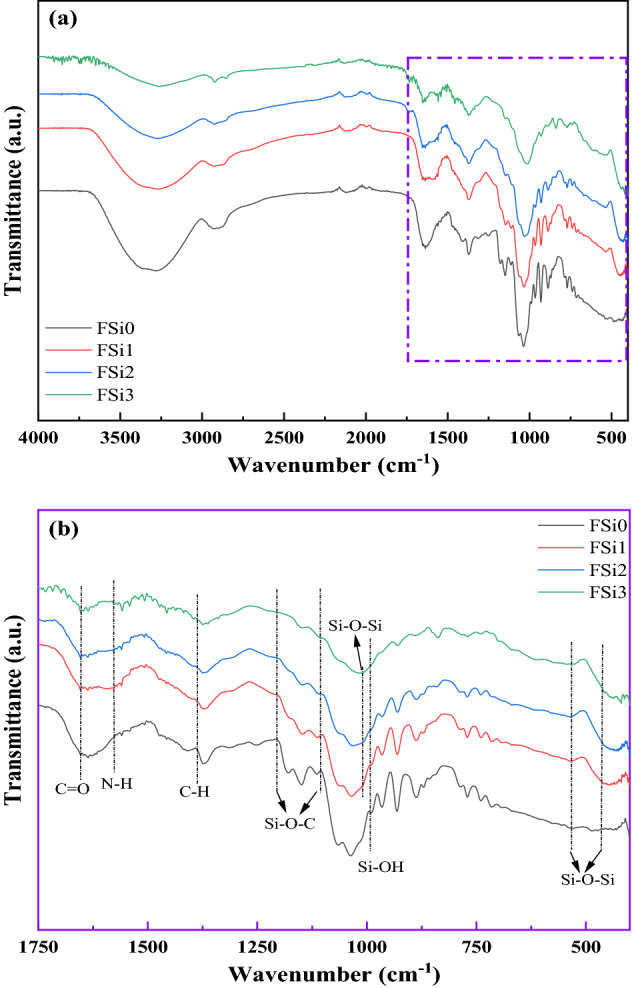
Fourier transform infrared spectroscopy (FTIR) of FSi group of samples (**a**) from 400 to 4000 cm^-1^; (**b**) from 400 to 1750 cm^-1^.

FTIR analysis was conducted to investigate the changes in chemical bonds due to the Si source and the interactions between fungi and PP fabrics. Figure 5 shows the transmission spectra of fungal fibers grown with different concentrations of Si source. Peaks in the spectral range at around 460 cm^-1^ and 540 cm^-1^ correspond to the bending and rocking of Si–O bonds respectively^23^. The broad band between 1000 and 1100 cm^-1^ was assigned to the stretching mode of Si–O and to the chemical constituents of fungal fibers, parts of which overlap^24^. The high bond energy of Si–O led to lower thermal conductivity and stronger fire resistance, delaying the degradation of fungal fibers^19^. The peak at around 960 cm^−1^, observed in samples containing Si source, was attributed to the silanol groups (Si–OH bond)^25^. The peak at 1386 cm^-1^ was attributed to C-H bending modes in FSi1, FSi2, and FSi3^26^. Meanwhile, an additional peak of N–H was detected at 1580 cm^-1^ and a free C = O bond was observed at around 1701 cm^-1^ in the FSi1, FSi2, and FSi3^27,28^. Their existence indicates that the structure of the fungal fibers changed due to the absorption of Si source. The bands at 1220 cm^-1^ and 1105 cm^-1^ were assigned to the Si–O–C in FSi1, FSi2, and FSi3^29,30^, which demonstrates that the Si source was digested by the fungal fibers and became part of their structure.

**Figure 6:**
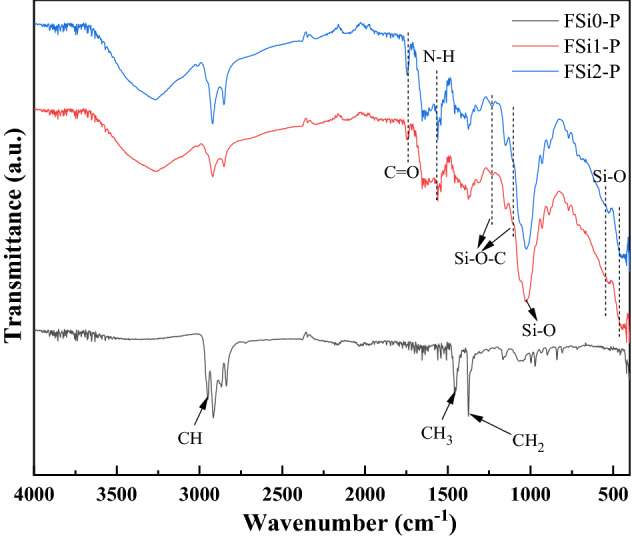
Fourier transform infrared spectroscopy (FTIR) results of the FSi-P group.

The FTIR analysis (Fig. 6) illustrates the representative infrared spectra of fungi-PP fabrics grown with different concentrations of Si source. The FTIR spectrum of FSi0-P was similar to that of PP fiber, from which the bands assigned to the CH, CH_2_, and CH_3_ were observed at 2950 cm^-1^, 1375 cm^-1^, and 1453 cm^-1^^31^. This indicates that the interaction between fungi and PP fabrics was physical, and that the fungal growth did not modify the chemical compounds of the PP fabrics. The absorption of Si source had a significant impact on the chemical functional groups of fungal fibers. The FTIR analysis on fungi-PP fabrics and fungi without PP fabrics, both grown with the same levels of Si source, yielded similar results. The bending and rocking of Si–O bonds generated bands from 460 to 540 cm^-1^. The band between 1000 and 1100 cm^-1^ was attributed to Si–O stretching vibrations^24^. Similar to the FSi group, C = O bond and N–H bond were found in the fungal fibers grown with Si source on PP fabric. The peaks at 1220 cm^-1^ and 1105 cm^-1^, observed in FSi1-P and FSi2-P, corresponded to the Si–O–C group^29,30^. Presence of these additional bands indicates that the absorption of Si source contributed to the formation of fungal cells in the fungi-PP fabrics samples.

#### Thermal stability analysis of fungi grown with different concentrations of Si source

**Figure 7:**
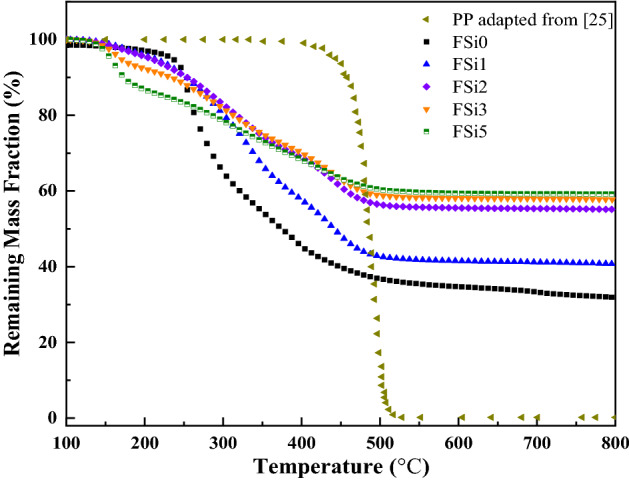
TGA of six fibers at a heating rate of 10 °C/min. TGA results and residual mass of PP fibers are adapted from ^32^.

Figure 7 shows the results of thermogravimetric experiments on *F. oxysporum* grown with different contents of Si source. It shows thermal degradation under a heating temperature range of 100–800 °C. The remaining weight curve of PP fibers was used as the baseline for the comparison with that of fungal fibers grown with different levels of Si source. The thermal parameters (*T*_onset_, the decomposition onset temperature; *K*_max_, the maximum mass loss rate; *T*_p_, the temperature at the maximum mass loss rate; *K*_ave_, the average thermal degradation rate; *T*_u_, the temperature at which residual weight is relatively stable; and the residual weight) were obtained from TGA and listed in Table 4.

**Table 4:** Summary of the results from TGA for fungal fibers grown with Si source as well as for the synthetic PP fibers.

Fiber type	*T*_onset_ (°C)	*T*_p_ (°C)	*K*_max_ (%/s)	*T*_u_ (°C)	*K*_ave_** (%/s)	Residue at 800 °C (wt%)
FSi0	250	264	0.137	500	0.033	31.94
FSi1	310	340	0.046	500	0.031	40.77
FSi2	310	340	0.028	500	0.022	55.15
FSi3	190	200	0.030	500	0.019	57.77
FSi5	160	170	0.052	500	0.014	59.20
PP*	468	489	42.50	520	0.270	0.20

*TGA results and residual weight of PP fibers are adapted from^32^.

**K_ave_, the average thermal degradation rate, is defined as the average mass loss rate per second in the range between *T*_onset_ and *T*_u_.

The TGA reveals that the decomposition onset temperature of sample FSi0 is 250 °C. The thermal degradation profiles of FSi1 and FSi2 show a similar trend with a higher decomposition onset temperature of 310 °C. However, the mass loss curves indicate an early decomposition, starting at 190 °C and 160 °C in FSi3 and FSi5, respectively. This is attributed to the absorption of relatively higher content of Si source (3% and 5% volume fractions) leading to a more soluble cell wall and an early loss of bonded water content from the cells^19^. With the increasing Si source in substrates, the fungal fibers became more thermally stable, as indicated by a smaller slope in the decomposition curves (i.e., smaller *K*_ave_, the average thermal degradation rate). The highest average thermal degradation rate of fungal fibers grown with different concentrations of Si source was found in FSi0 (0.033%/s), which is about one tenth of that of PP fibers (0.270%/s). The absorption of Si source also contributed to an improvement of residual weight in fungal fibers. More residuals were retained in the fungal fibers grown with higher contents of Si source, and the residual weights of FSi0, FSi1, FSi2, FSi3, and FSi5 were 31.94%, 40.77%, 55.15%, 57.77%, and 59.20%, respectively. The char yield was more sensitive to the Si source at small concentrations (i.e., below 2%). When the Si source concentration was higher than 2%, the residual weight was around 55–59% and only a minor increase occurred with the Si source concentrations.

The TGA results of PP fibers were adapted from^32^ and used to compare of fungal fibers grown with various concentrations of Si source. The decomposition temperature in fungal fibers grown with Si source ranged from 160 to 310 °C, which is much lower than that of PP fibers. This would contribute to earlier melting of fibers that creates voids in concrete, releases temperature-related vapor stress, and potentially improves the fire safety of concrete structures. Meanwhile, the maximum mass loss rate and average degradation rates of fungal fibers grown with Si source are much smaller than that of PP fibers. The smaller thermal degradation rate of fungal fibers improves the thermal stability over PP fiber when exposed to high temperatures. Additionally, fungi fibers achieved high residual weights (31.94–59.20%), which are significantly larger than that of PP fibers (only 0.2%) after exposure to a temperature of 800 °C.

#### Flammability analysis by MCC

**Figure 8:**
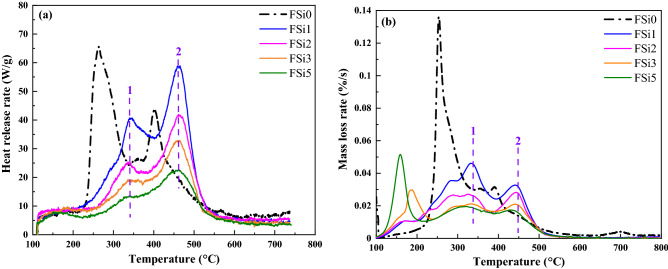
(**a**) The heat release rate at different temperature for fungal fibers grown with different concentration of Si source in the MCC test; (**b**) the calculated mass loss rate for fungal fibers grown with different concentration of Si source from the TGA test.

MCC is a standard method for fire-resistant assessment. It measures the heat release by the combustion of the vapor released from the material at an increasing temperature. The MCC results from fungal fibers grown with Si source were compared with fungal fibers grown with Si-free substrate. The heat release rates at different temperatures for each sample are shown in Fig. 8a. The mass loss rate curves from TGA are summarized in Fig. 8b. Compared with the fungi fibers grown with Si-free substrate (FSi0), the addition of Si source in the substrate retarded the release of combustible vapor in mycelium fibers. The most prominent peaks of heat release rate in FSi0 were at 250 °C and 400 °C. In comparison, these peaks increased to higher temperatures (i.e., 340 °C and 460 °C) in the fungal fibers grown with Si source (i.e., FSi1, FSi2, FSi3, and FSi5). The release heat rate decreased in the fungal fibers grown with higher concentrations of Si source since the peak temperature was lower in the samples grown with higher concentration of Si source.

The observed peaks at 250 °C and 400 °C in the mass loss rate curve from TGA of FSi0 are consistent with the peaks shown by MCC measured heat release rate curves. This indicates that the mass loss in TGA at these peaks are associated with the release of combustible vapor at 250 °C and 400 °C. For fungal fibers grown with Si source, the release of combustible mass leads to peaks at 340 °C and 450–460 °C, which is much higher than that of fungi fibers grown without Si source. Moreover, with the increase of Si source, the mass loss rate decreased in the range from 250 to 500 °C, which is consistent with the trend seen in the MCC measured heat release rate curves. However, additional peaks were observed from 150 to 250 °C in the TGA mass loss rate curves of FSi3 and FSi5, while no peaks presented at the same locations in the MCC heat release rate curves. This implies that the vapor in the range of 150 °C and 250 °C was non-combustible. A possible explanation is that when grown with higher concentration of Si source, the cell walls of fungal fibers become more flexible, which leads to the release of more non-combustible vapor at lower temperatures (150–250 °C) and subsequently the less release of combustible vapor at higher temperatures (250–500 °C).

**Figure 9:**
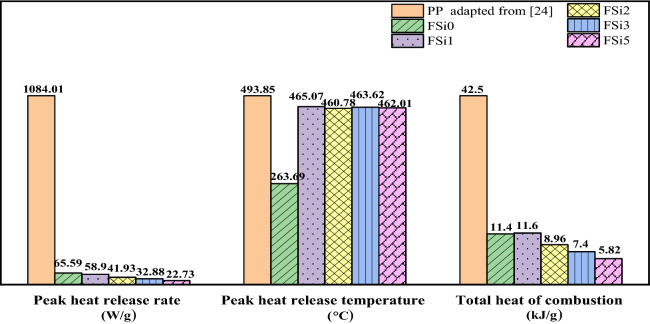
The peak heat release rate, peak heat release temperature and total heat of combustion of fungal fibers grown with different contents of Si source as well as PP fibers.

The peak heat release rates and the total heat of combustion are summarized in Fig. 9. The peak heat release rates of fungal fibers were 65.59 W/g, 58.90 W/g, 41.93 W/g, 32.88 W/g, and 22.73 W/g in FSi0, FSi1, FSi2, FSi3, and FSi5, respectively. The peak heat release rate was reduced up to 66% by the addition of 5% Si source in the feeding substrate. The heat release rate of PP fibers was 1084.01 W/g, which is 47 times higher than that of FSi5. The peak heat release temperature of FSi0 was 263.69 °C. The temperatures corresponding to the peak heat release rate of fungal fibers were 465.07 °C, 460.78 °C, 463.62 °C, and 462.01 °C in FSi1, FSi2, FSi3 and FSi5, respectively. The heat release peak temperature of PP fibers was 493.85 °C^32^. The total heat of combustion of FSi0, FSi1, FSi2, FSi3 and FSi5 were 11.4 kJ/g, 11.6 kJ/g, 8.96 kJ/g, 7.40 kJ/g and 5.82 kJ/g, respectively. This indicates that Si source in the feeding substrate contributed to the reduction of the total heat of combustion. The total heat of combustion of PP fiber was 42.50 kJ/g^32^, which is seven times higher than that of FSi5.

Overall, the results of MCC indicate that grown with Si source increased the threshold temperature for the release of combustible vapor in the fungal fibers; it also reduced the amount of combustible vapor released and combustion heat in the fungal fibers. Both of these factors improve the fire resistance of Si-sourced fungi fibers. Fungal fibers grown with Si source have significantly lower peak heat release temperature as well as lower total heat of combustion, which contributes to the improved fire safety. Compared with the commonly used PP fibers, these features of fungi fibers will help improve fire resistance when used in fiber reinforced concrete.

### Lab-scale characterization

#### Heating ignition test

The heating ignition test was used to evaluate the fire resistance of the materials at laboratory-scale. Fire resistance was characterized by time to ignition, temperature when ignition, height of flame, and occurrence rates of ignition. Ignition time was defined as the moment at which a flame appears on the surface of samples. Ignition temperature was measured at the time at which ignition occurs. Flame height was characterized as the maximum height the flame reached. Ignition Occurrence Rate equals the number of samples that were ignited before they were completely degraded divided by the total number of samples. However, in some samples, ignition was never achieved. The ignition time, ignition temperature, and Ignition Occurrence Rate characterized the fire resistance of the fungal fibers while the flame height indicated the flaming intensity once ignited. Fungal fibers grown with 0%, 1%, and 2% Si source were tested and compared with PP fabrics. Fungi fibers grown with 3% and 5% exhibited growth rates so slow that they were not considered in the following laboratory-scale fire resistance tests.

**Figure 10:**
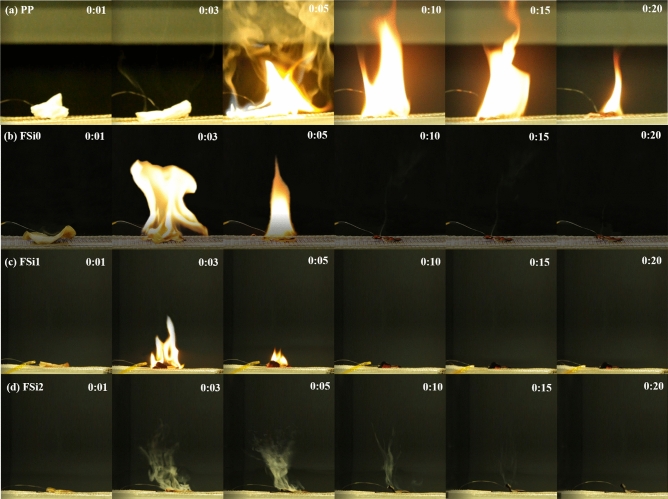
Video screenshots of representative samples (**a**) PP; (**b**) FSi0; (**c**) FSi1; (**d**) Ignition did not achieve in FSi2 during the heating ignition test.

As revealed by the heating ignition test (Fig. 10), samples became crimped once they were heated. Ignition occurred in the first 3–5 s in the PP sample, FSi0 sample, and FSi1 sample. However, ignition and flame were never reached in FSi2, whose whole sample was degraded by heating. A wide and high flame with thick smoke was found in the PP sample. The addition of Si source in the feeding substrate decreased the intensity of fire in the fungal samples. The only thing observable in FSi2 is smoke.

**Figure 11:**
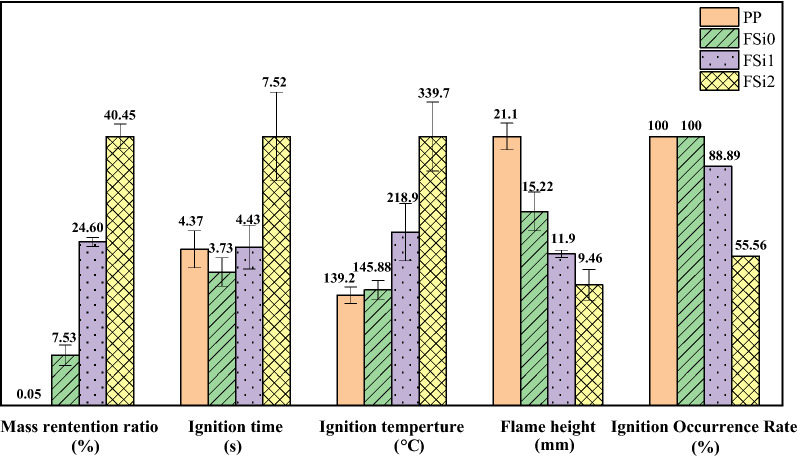
The heating ignition test results for fungal fibers grown with different concentrations of Si source.

As shown in Fig. 11, the cultivation with Si source increased the mass retained in the fungi sample. The mass retention ratio increased by 17.07% and 32.92% when comparing fungal fibers grown without Si source to those grown with 1% and 2% Si source. Meanwhile, PP fabrics burnt almost completely and only 0.05% were retained after the test. Most compositions in PP fabrics are combustible, which explains the long ignition persistence in PP fabrics (shown in Fig. 10a). Time to ignition of PP fabrics was measured as 4.37 s, and ignition time in FSi2 was 7.52 s. Ignition temperature, flame height, and Ignition Occurrence Rate are key factors in a fire accident. The flame height of fungal fibers (FSi0) was 5.88 mm lower and the ignition temperature was 6.88 °C higher than PP fibers. The fungal fibers cultivated with 1% and 2% Si source also caused a significant increase in the ignition temperature (by 73.02 °C and 193.82 °C) and a decline in flame heights (by 3.32 mm and 5.76 mm). One out of nine fungal samples grown with 1% Si source and four of nine fungal samples grown with 2% Si could not be ignited or degraded without flaming. These qualities indicate that fungal fibers achieved higher fire resistance than PP fibers by reducing the possibility of ignition and weakening the flames; higher concentrations of Si source in feeding substrate further improved the fire resistance of fungal fibers.

#### Upward flame spread test

**Figure 12:**
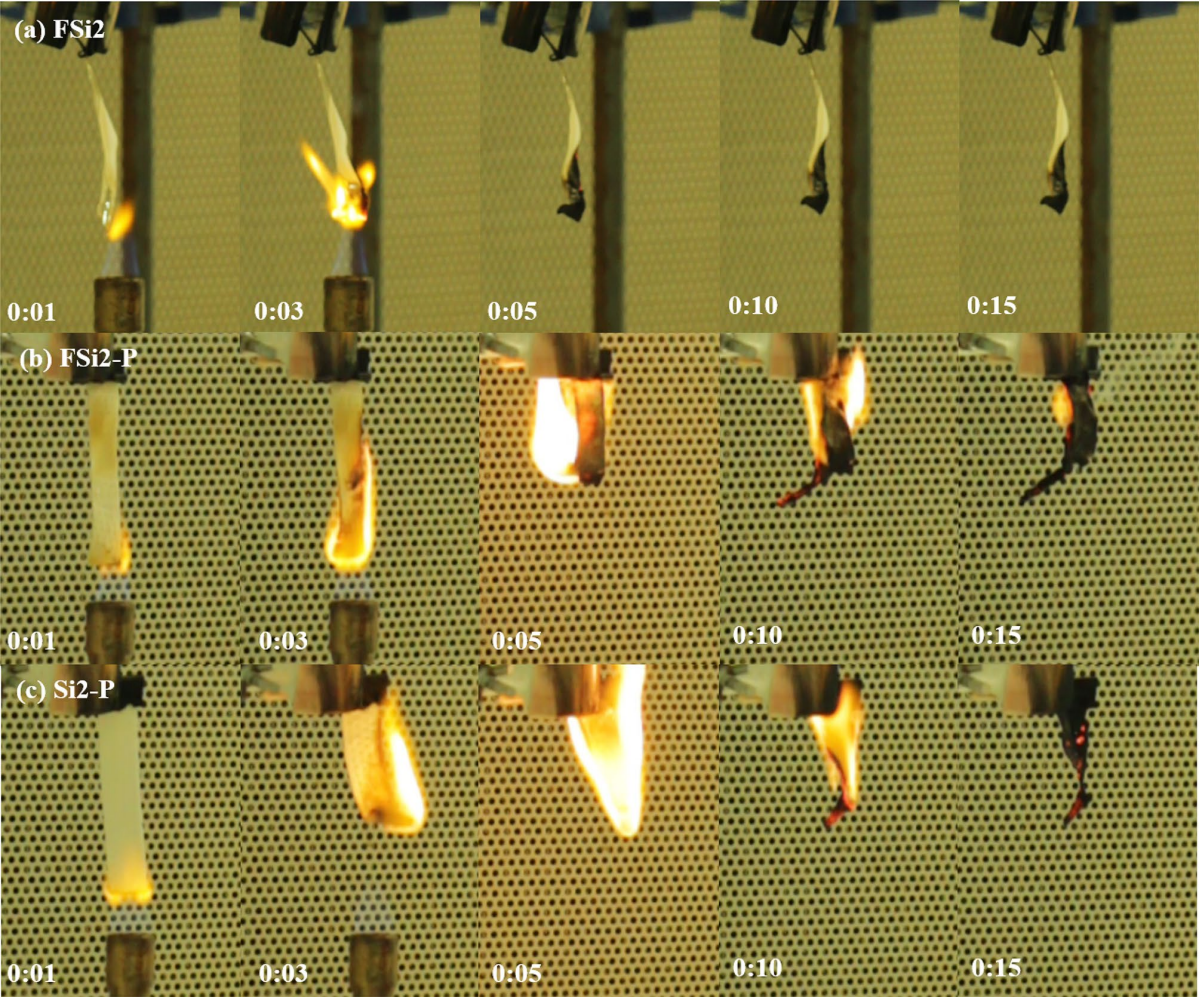
Video screenshots of representative samples (**a**) Partial burning in FSi2 (fungal fibers grown with 2% Si source); (**b**) FSi2-P (fungi-PP fabrics grown with 2% Si source); (**c**) Si2-P (PP fabrics submerged in substrate with 2% Si source) during the upward flame spread test.

The upward flame spread test modified from the UL-94 V test was applied to characterize the flame spread rate of fungal materials grown with various Si source. A thin film grown by fungal fibers (FSi0, FSi1, and FSi2) were prepared as thin bar samples with a thickness of 0.25 mm. Fungi-PP fabrics (FSi0-P, FSi1-P, and FSi2-P) were also prepared as bar samples with a thickness of 0.65–0.70 mm. Additionally, PP fabrics (Si0-P, Si1-P, and Si2-P) with a thickness of 0.65–0.70 mm were submerged in the substrate containing different concentrations of Si source for the same incubation period to exclude the influence of Si source on the measurement of flame spread rate. The upward flame spread tests were undertaken using the conditions as described in Sect. “Upward flame spread test”. The observed flame spread phenomena of the contrast samples are shown in Fig. 12.

The Fully Burnt Rate was calculated by the number of samples that were burnt completely divided by the total number of samples. In the FSi (fungi grown in substrate with different Si source concentrations) and FSi-P (fungi-PP fabrics grown with different Si source concentrations) group, not all bar samples were fully burnt; some had residual unburnt material. The Fully Burnt Rates are summarized in Table 5. All samples in the Si-P (PP fabrics submerged in substrate with different Si source concentrations) group were completely burnt, while fungal fibers grown with 1% and 2% Si source were not fully consumed. This indicates that the biological absorption of Si source by fungal metabolism is more effective than the physical absorption of Si source in the Si-P group.

**Table 5:** Fully Burnt Rate in the upward flame spread test.

	FSi0	FSi1	FSi2	FSi0-P	FSi1-P	FSi2-P	Si0-P	Si1-P	Si2-P
Si content	0%	1%	2%	0%	1%	2%	0%	1%	2%
Fully Burnt Rate (%)	100.00	77.78	22.22	100.00	100.00	88.89	100.00	100.00	100.00

From Fig. 12b, c, it is clear that once FSi2-P and Si2-P were ignited, they burned quickly, and the flame in Si2-P was brighter and stronger than that in FSi2-P. The fungi-PP fabrics grown with 2% Si source were more fire-resistant than PP fabrics submerged in 2% Si source. This indicates that the fungal fibers decreased the intensity of the flame in the PP fabrics. In particular, the fungal fibers grown with 2% Si source were observed to have high fire-resistance. Once the burner was removed, flame in the FSi2 disappeared (Fig. 12a).

Based on the recorded mass and flame time in the upward flame spread test, mass retention ratio was defined as the final mass divided by the original mass of each sample; flame spread rate was defined as the mass loss ratio divided by total flame time.

**Figure 13:**
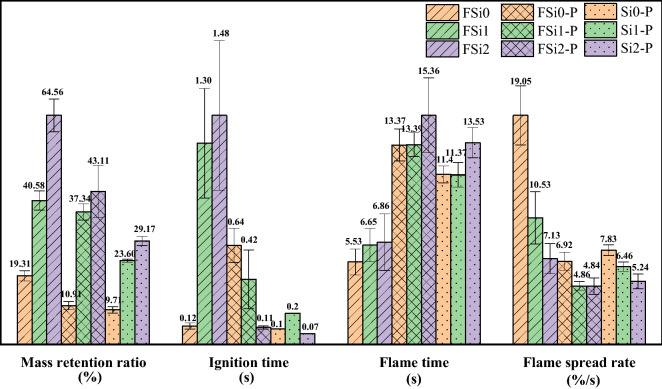
Upward flame spread test results for fungal fibers grown with Si source.

Figure 13 shows the results of the upward flame spread test in three groups, i.e., FSi, FSi-P, and Si-P. In the FSi group, with the addition of 1% and 2% Si source in the substrate, 983.33% and 1133.33% longer ignition time, and 20.25% and 24.05% longer flame time were observed in the upward flame spread test; 110.15% and 234.33% more mass was retained in the residual samples after the tests. The flame spread rates of samples decreased by 44.72% and 62.57% in the FSi1 and FSi2. All fungal fibers grown without Si source were completely burnt, however, the Fully Burnt Rates of fungal fibers grown with 1% and 2% Si source were lower (FSi1 was 77.78% and FSi2 was 22.22%).

In the FSi-P group, mass ratios retained in FSi1-P and FSi2-P were 242.56% and 295.14% higher than that retained in FSi0-P. The flame time was increased up to 14.88% in FSi2-P, compared with FSi0-P. The flame spread rate was also decreased by 29.77% and 30.06% for fungi fibers grown with 1% and 2% Si source. All fungi-PP fabrics grown with 0% and 1% Si source were completely burnt. Partial burning was observed in fungi-PP fabrics grown with 2% Si source with a Fully Burnt Rate of 83.33%.

In the Si-P group, 143.05% and 200.41% higher mass retention ratios were obtained in Si1-P and Si2-P, compared with that in Si0-P. The flame time in Si2-P was 18.68% longer than that in Si0-P. The absorption of 1% and 2% Si source led to the reduction of flame spread by 17.50% and 33.08%. All of the samples in the Si-P group were burnt completely.

Among the three groups, more mass was retained in the samples with increasing Si source concentrations in the feeding substrate. The mass retained after the upward flame spread test increased with the increase of Si source and the mass retention ratio increased by 45.25%, 32.20%, and 19.46% when adding 2% Si source in the FSi, FSi-P, and Si-P group. Comparing the increased mass retention ratio in the FSi and Si-P group, it can be concluded that the higher mass retention ratio in the FSi-P group mainly caused by the grown fungi fibers rather than the addition of Si source. The larger mass retained in the residual sample (char yield) was a significant indicator of improved fire resistance, which can potentially improve fire safety when used as fiber reinforcement for concrete structures. The ignition time was longer in the samples in the FSi group than the corresponding samples in the Si-P group with the same concentration of Si source, which shows that fungal fibers are much more difficult to ignite than the PP. The flame spread rates in FSi-P and Si-P groups were similar, which shows that fire spread on flammable PP fibers determines how fast the fire will spread on fungi-PP fabrics. Flame spread rates were significantly reduced in the FSi group, and flame spread rates were further reduced with the increase of Si source. The spread of fire was delayed by the increasing of char that impeded the release of combustible vapor and heat transfer. As shown in Fig. 13 and Table 5, the reduction of the Fully Burnt Rates is caused by the increase of residual mass (or char yields). Additionally, as the MCC results show, the combustible heat release was reduced in fungi grown with increasing Si source, which also contributes to the decrease of the Fully Burnt Rate.

### Characterization in post-fire conditions

#### SEM/EDS analysis of char

**Figure 14:**
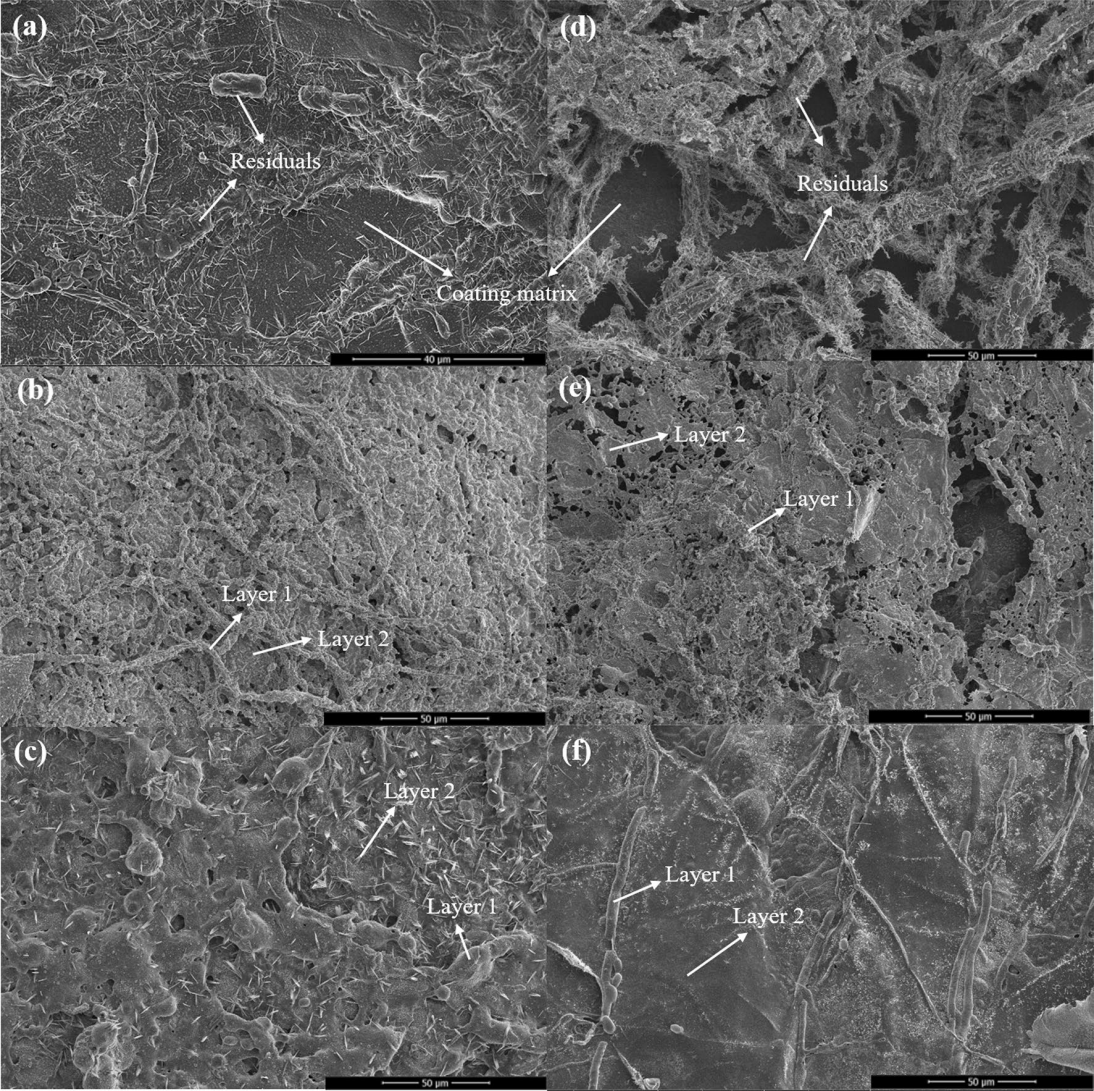
Micrographs of residual chars from (**a**) FSi0, (**b**) FSi1, (**c**) FSi2, (**d**) FSi0-P, (**e**) FSi1-P, and (**f**) FSi2-P.

The morphology of char has a significant impact on the fire-resistance. The residual chars were obtained from the burnt samples after the upward flame spread test and their microstructures were characterized by SEM. Figure 14a–c presents the microstructures of the chars obtained from FSi0, FSi1, and FSi2, respectively. Sparse residuals were observed in FSi0 and condensed residuals were retained in samples with higher concentrations of Si source. The microstructure of chars from FSi2 appeared as dense as an intact layer. This condensed char layer blocked both the fuel vapor release and heat transfer to the sample. Similar conclusions were obtained through the analysis of the fungi-PP fabrics grown with Si source after an upward flame spread test (Fig. 14d–f). Figure 14e and f shows that the microstructure of char from FSi1-P and FSi2-P became denser compared with that obtained from FSi0-P. Si source served as a crosslinker in fungal fibers’ structure and contributed to the low thermal conductivity as well as to the delay of vapor generation. This resulted in a dense structure that enhanced the fire protection capability of the residual chars. It is clear that PP fabrics were susceptible to fire because only the fungal fibers and their surroundings were present in the char of FSi0-P. This indicates that fungal fibers have higher resistance to fire than PP fibers. With the increasing Si content in samples, a larger surrounding area could be retained along the fungal fibers. This demonstrate that absorption of Si source further improved the fire resistance of fungal fibers.

**Figure 15:**
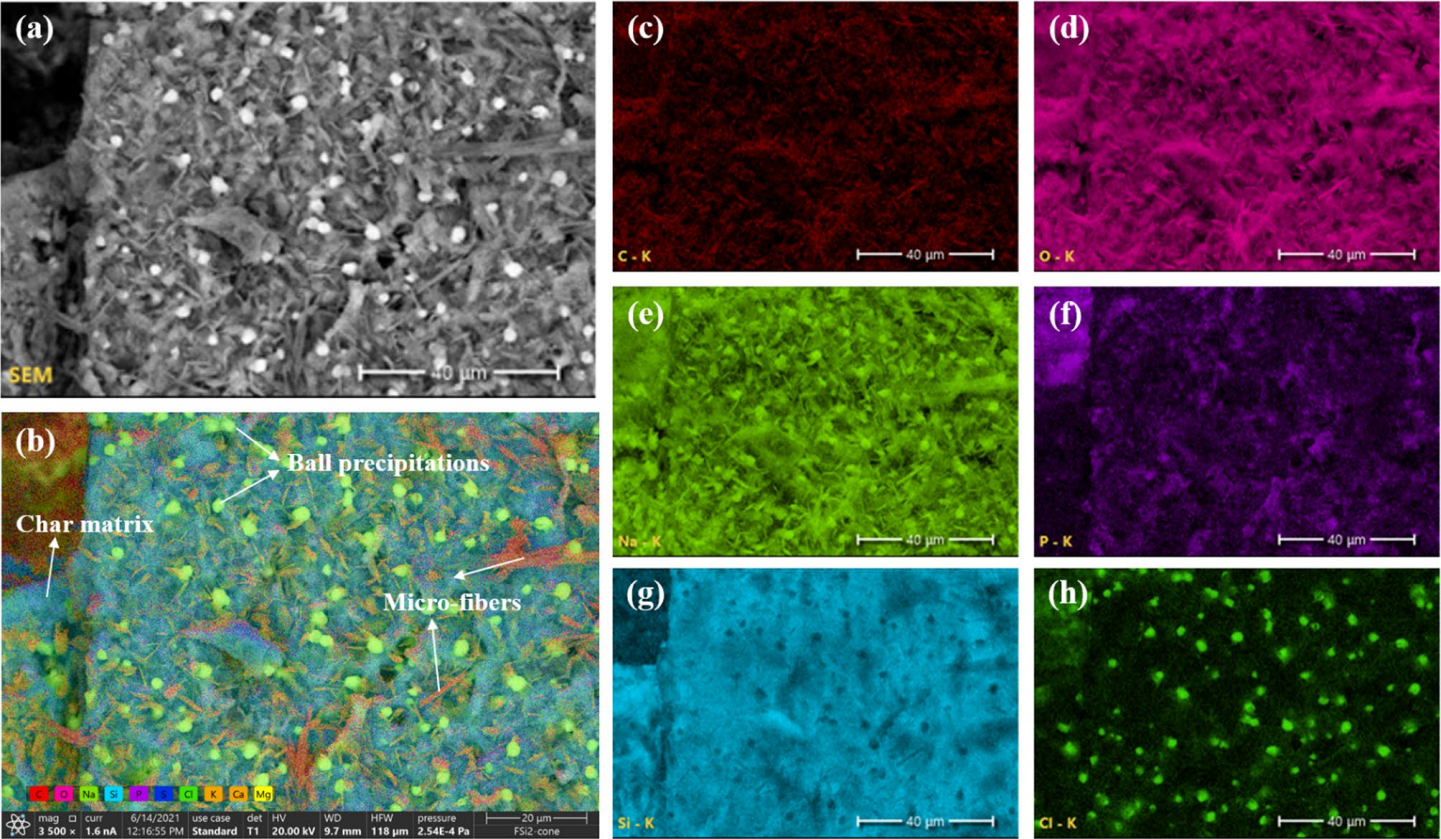
EDS analysis on FSi2 sample (**a**) selected area on FSi2, (**b**) main element mapping on surface, (**c**) element mapping of C, (**d**) element mapping of O, (**e**) element mapping of Na, (**f**) element mapping of P, (**g**) element mapping of Si, (**h**) element mapping of Cl.

To further understand the chemical composition of char, EDS analysis was conducted on the chars from fungal fibers grown with different concentrations of Si source. Figure 15 shows the main element mappings in char samples obtained from fungal fibers grown with 2% Si source (FSi2) after the heating ignition test. Numerous microfibers and ball precipitations were observed on the surface of the char. The individual element mappings (Fig. 15c–h) demonstrate that the main compositions of the microfibers were carbon and sodium, and the main composition of the ball precipitations was sodium and chlorine. Silicon was not included in the microfibers and ball precipitations. The main fiber structures were destroyed after being exposed to fire, and carbon microfibers were retained. Soluble fungal structure accelerated the evaporation of water contents originally contained in the fungal fiber cell when grown with Si source. The salty composition (sodium and chlorine) and Si source spread from the fungal cells and were found all over the char. The weight ratios of the main elements were summarized in Table 6. With the increase of Si source in the feeding substrate, more Si content was retained after burning. The higher content of Si source slightly enhanced the production of carbon char, which was demonstrated by the carbon contents being 7.9%, 8.7%, and 12.6% in the residuals of samples from FSi0, FSi1, and FSi2, respectively. These traces indicate that Si source that was absorbed in fungal cells improved the char production, which is a significant indicator of stable structure under fire.

**Table 6:** The weight ratio of the main elements in residual samples.

Sample ID	Si content (%)	C	O	Na	P	S	Cl	K	Ca	Si
FSi0	0	7.9	43.4	23.1	7.7	3.8	2.7	8.0	1.7	0.1
FSi1	1	8.7	44.9	23.5	5.0	0.1	0.1	1.4	0.1	16.0
FSi2	2	12.6	46.0	12.8	0.7	1.1	0.6	1.2	0.2	24.5

#### FTIR analysis

**Figure 16:**
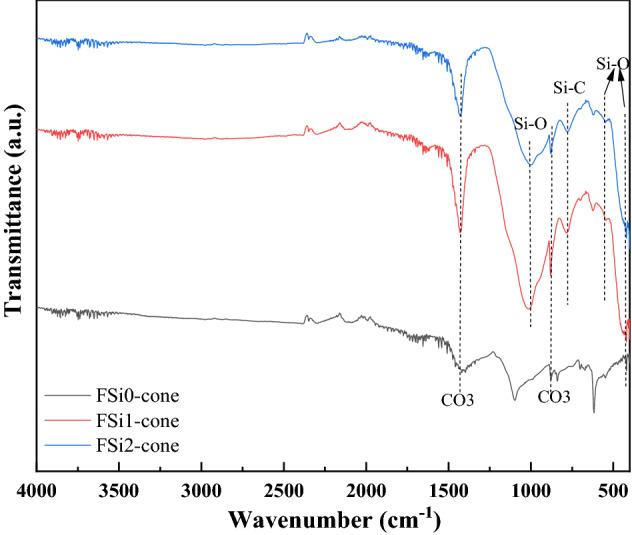
The FTIR results of samples in the FSi group subjected to fire.

The Fourier transform infrared spectroscopy (FTIR) analysis of residual char samples after the heating ignition test are shown in Fig. 16. The Si–O–C, Si–OH bonds were converted into Si–O bonds during burning^33^. The Si–O bond was stable and its stretching, bending, and rocking vibration were detected between 1000 and 1100 cm^-1^, at 460 cm^-1^, and at 540 cm^-1^ in the residual char in the fungal fibers grown with Si source^23,24^. The band at 770 cm^-1^ corresponded to the presence of Si–C bond in the residual char^34^. Si–O and Si–C bonds were only detected in the char from the fungal fibers grown with Si source. The sharp band at 879 cm^-1^ corresponded to the symmetric deformation of the CO_3_ group^35^. The broad band at 1430 cm^-1^ was also attributed to the formation of the CO_3_ group^36^. The CO_3_ group was found in all these samples and was formed by the organic components of fungi exposed to high temperatures. Si–O and Si–C chemical bonds formed in the fungal fibers grown with Si source have higher heat of chemical bond formation (452 kJ·mol^−1^ and 360 kJ·mol^−1^) compared with the C–O bond (358 kJ·mol^−1^) in fungal fibers grown without Si source^37^. The formation of Si–O and Si–C prevents further pyrolysis and the release of combustible gases, which reduced the heat release rate of fungi grown with Si source^19^. They contributed to the excellent thermal and fire resistance of fungal fibers^38,39^.

**Figure 17:**
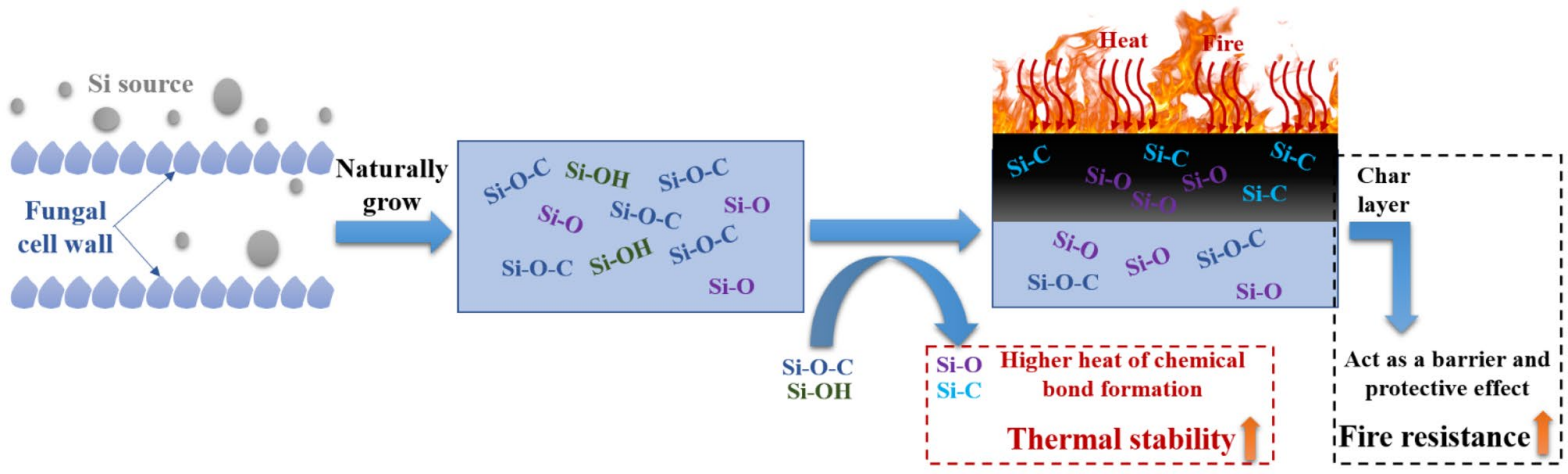
Mechanism of thermal stability and fire resistance of fungal fibers grown with Si source.

In summary, fungal fibers grown with Si source are highly thermally stable and fire resistant. The schematic of the mechanism for enhanced fire resistance with Si is shown in Fig. 17. Si source firstly entered fungal cell , leading to the formation of Si–O–C, Si–OH, and Si–O bonds in fungal fibers. When exposing to fire and heat, Si–O–C and Si–OH bonds in fungal fibers were converted into Si–O bond and Si–C bond was also generated in burning fungal fibers. Si–O and Si–C have higher heat of chemical bond formation, contributing to the improved thermal stability. Denser char layer was produced in fungal fibers grown with Si source during fire incident, acting as a barrier to prevent the release of combustible vapor. This would enhance fire resistance of fungal fibers.

## Conclusion

A fungal strain, *F. oxysporum*, was cultivated with Si source to produce natural fire-resistant fibers. The fire resistance of fungal fibers was investigated by experiments conducted at the micro-scale and laboratory-scale. The characterization was also conducted on samples in post-fire conditions. The following conclusions are drawn:

1. At micro-scale, scanning electron microscopy images show that the surface of fungal fibers grown with 0%, 1%, and 2% Si source was straight and continuous. Grown with higher concentrations of Si source, fungi fibers had a segmented structure and a larger diameter. Fourier-transform infrared (FTIR) spectra show the existence of Si–O-C chemical bond in fungal fibers grown with Si source, indicating that Si source was incorporated as part of the fungal structures. Thermogravimetric analysis (TGA) and Microscale combustion calorimetry (MCC) of fungal fibers show an early thermal decomposition of non-combustible components, which contributes to the early release of thermal stress and to the mitigation of concrete spalling. Fungal fibers had a lower thermal degradation rate, a higher residual weight, a lower heat release peak temperature, and less total heat of combustion compared with PP fibers, which indicates a lower rate of function loss and an improvement of fire resistance. Additionally, the thermal stability and fire resistance of fungal fibers were further improved with the increase of Si source in the feeding substrate.
2. At laboratory-scale, fungal fibers demonstrate a better performance in fire resistance than PP fibers in real fire scenarios. Fungal fibers retained more weight and had a slower flame spread rate in the upward flame spread test; fungal fibers also had a higher ignition temperature and a smaller flame height in heating ignition test. The absorption of Si source is believed to improve the fire resistance of fungal fibers. Additionally, partial burning only occurred in fungal fibers grown with Si source.
3. In post-fire conditions, the microstructure of residual char from fungal fibers grown with a higher Si source became denser. This is believed to reduce the fuel vapor release and heat transfer. FTIR spectra of char samples show that the absorption of Si source in fungal fibers structure formed chemical bonds with higher heat of chemical bond formation such as Si–C, contributing to its large char yield and excellent fire resistance.

Overall, the multi-scale characterization demonstrates that fungal fibers grown with silica source achieved much higher fire resistance than tradition PP fibers that are commonly used for fiber reinforced concrete to mitigate shrinkage cracks etc. The experimental evidence showed that Si source in the feeding substrate was utilized to establish the structure of fungal fibers, which contribute to the significant improvement of thermal stability and fire resistance. The high thermal stability of fungal fiber can potentially improve concrete fire resistance by mitigating concrete spalling due to high temperature and improve fire safety of concrete structures.

## Data Availability

The datasets generated and/or analyzed during the current study are not publicly available due but are available from the corresponding author on reasonable request.

## List of symbols

*T*_onset_: Decomposition onset temperature (°C)
*T*_p_: Temperature at the maximum mass loss rate (°C)
*T*_u_: Temperature at which residual weight is relatively stable (°C)
*K*_max_: Maximum mass loss rate (%/s)
*K*_ave_: Average thermal degradation rate (%/s)
